# Exercise, cancer, and the cardiovascular system: clinical effects and mechanistic insights

**DOI:** 10.1007/s00395-024-01034-4

**Published:** 2024-02-14

**Authors:** Simon Wernhart, Tienush Rassaf

**Affiliations:** West German Heart- and Vascular Center, Department of Cardiology and Vascular Medicine, University Hospital Essen, University Duisburg-Essen, Hufelandstrasse 55, 45147 Essen, Germany

**Keywords:** Exercise, Cancer, CPET, CRF

## Abstract

Cardiovascular diseases and cancer are the leading causes of death in the Western world and share common risk factors. Reduced cardiorespiratory fitness (CRF) is a major determinant of cardiovascular morbidity and cancer survival. In this review we discuss cancer- induced disturbances of parenchymal, cellular, and mitochondrial function, which limit CRF and may be antagonized and attenuated through exercise training. We show the impact of CRF on cancer survival and its attenuating effects on cardiotoxicity of cancer-related treatment. Tailored exercise programs are not yet available for each tumor entity as several trials were performed in heterogeneous populations without adequate cardiopulmonary exercise testing (CPET) prior to exercise prescription and with a wide variation of exercise modalities. There is emerging evidence that exercise may be a crucial pillar in cancer treatment and a tool to mitigate cardiotoxic treatment effects. We discuss modalities of aerobic exercise and resistance training and their potential to improve CRF in cancer patients and provide an example of a periodization model for exercise training in cancer.

**Supplementary Information:**

The online version contains supplementary material available at 10.1007/s00395-024-01034-4.

## Introduction

Cardiovascular diseases and cancer are the leading causes of death in the Western world and share common risk factors, such as smoking, arterial hypertension, diabetes, sedentary lifestyle, aging, unbalanced diet, and alcohol consumption [122]. In 2022, almost 2 million new cancer cases and more than 600.000 cancer deaths were registered in the USA, including approximately 350 deaths per day from lung cancer, which is the leading cause of cancer death [182]. In 2020 breast cancer was the world´s leading cause of cancer diagnosis with 2.26 million cases [216]. Apart from interventions to reduce common risk factors, the World Health Organization recommends physical activity on a regular basis [216]. As the COVID-19 pandemic has set back cancer research and treatment progress, the Lancet Oncology European Groundshot Commission was founded, which created an action plan aiming to achieve a 10-year survival for 70% of all European cancer patients by 2035 [109]. In this review, we discuss the cardiovascular risk of cancer patients as well as cancer treatment-related cardiotoxicity. We highlight limitations in cardiorespiratory fitness (CRF) induced by parenchymal, cellular, and mitochondrial dysfunction. Mechanisms of cancer-related disruption of signaling and metabolic pathways, which culminate in impaired energy expenditure, are illustrated. Cardiopulmonary exercise testing (CPET) may present a promising diagnostic approach to tailor exercise programs designed to antagonize those effects on a molecular and macroscopic level. We performed a systematic research in PubMed® using the terms “exercise” AND “cancer” as well as “sports” AND cancer “ to search for clinical trials, randomized controlled trials, and meta-analyses. Clinical, translational, and basic science articles were screened and those with objective documentation of exercise intervention or physical activity (PA) were included in this review.

## Cardiovascular risk and cardiotoxicity in cancer patients

Patients suffering from cancer bear a higher risk for the development of cardiovascular diseases compared to age-matched controls [122]. Although treatment options of various cancer types have improved over the last decade, specific agents used in cancer therapy increase the probability to develop cardiovascular diseases, arterial hypertension, hypercholesterolemia, diabetes, and may increase body weight [39, 47] as well as the incidence of cardiovascular adverse events [141].

Cancer therapies, especially breast cancer treatment with anthracyclines, increase the risk to develop heart failure [2]. Breast cancer survivors display a higher risk of cardiovascular death eight years after diagnosis [162]. Early initiation of heart failure therapy in breast cancer patients with cardiotoxicity due to anthracycline therapy has been shown to be able to recover left ventricular function [147].

Chemotherapy as well as antiangiogenic drugs have been associated with an increased risk of adverse cardiac events: Bevacizumab has been associated with hypertension, anthracyclines with heart failure and left ventricular dysfunction, taxanes with arrhythmias, and antimetabolites with thromboemboli and spasms [122, 141]. Certain drugs predispose to pulmonary hypertension, such as tyrosine kinase inhibitors and alkylating agents, or increase the risk of ischemic events and arterial hypertension (bevacizumab), which can increase the risk to develop heart failure [79, 194, 195]. Immune checkpoint inhibition targeting programmed cell death 1 disrupts cardiac immune function and may lead to immune mediated myocarditis [102, 133, 137], while chimeric antigen receptor—T cell therapy can trigger cytokine release syndrome, which facilitates cardiovascular events [191, 192]. Specific ECG screening prior to chemotherapy [158] as well as biomarkers such as NTproBNP and troponin and approaches in echocardiography have been regarded as valuable markers to detect apparent and sub-clinical cancer therapy-related cardiotoxicity [73, 134–136]. Similar data are available for nuclear and molecular imaging techniques [93, 190]. In more compromised patients with advanced cancer stage the self-reported ability to walk for 4 minutes and wash oneself were independent predictors of survival [11].

Cancer patients are a high-risk population, in which long-term mortality following cardiological interventions is higher compared to non-cancer patients [89, 116, 143, 187] and procedures may even be denied due to shortened life expectancy [43, 116]. Low left ventricular mass and reduced handgrip strength reflect lower functional status and increased all-cause mortality in patients with cancer without manifest cardiovascular disease [65, 114]. Due to the complexity of these patients, multi-disciplinary cardio-oncology teams should be established to optimize preventive and therapeutic measures [196] and further trials to investigate benefits of cardio-protective drugs in cancer patients are still warranted [193].

**Fig. 1:**
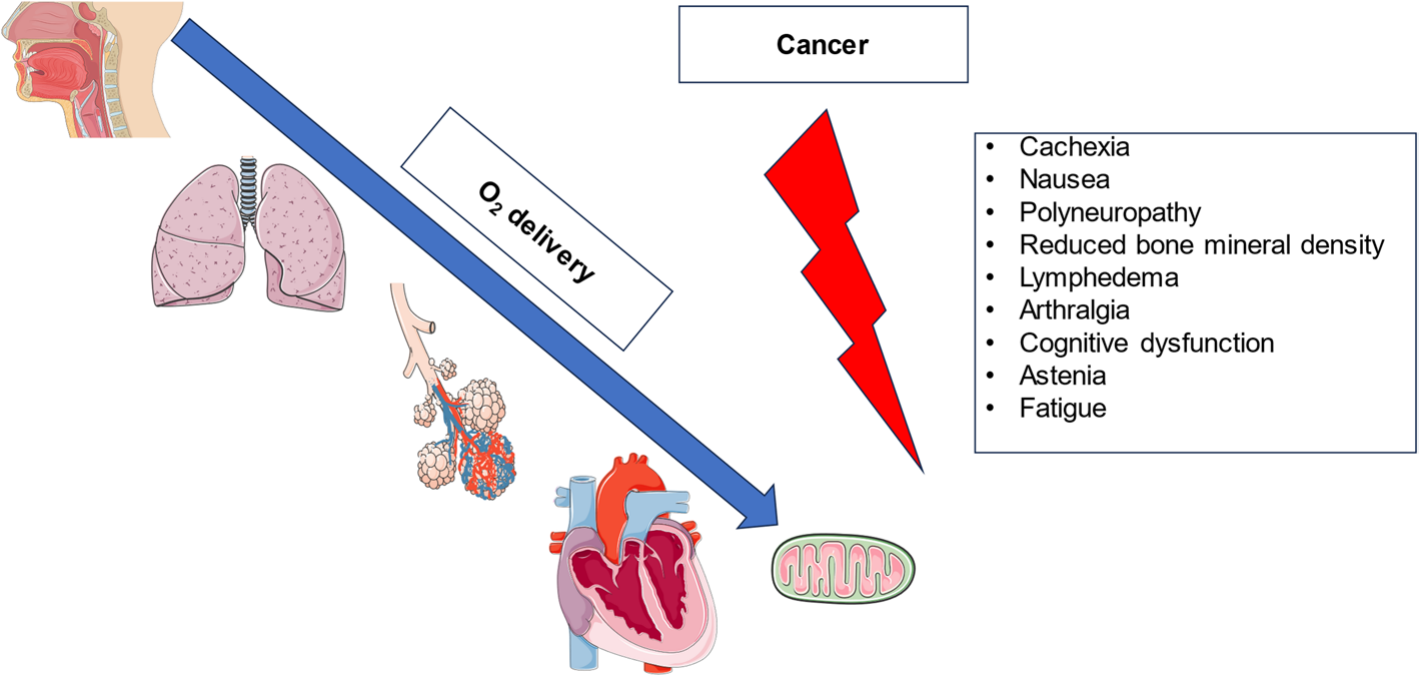
Limitations of oxygen transfer from the upper airways to the mitochondrion in cancer patients and associated clinical symptoms. Created with BioRender.com

Although cardio-oncology has been introduced as a sub-discipline to investigate cardiotoxic effects of cancer itself and associated therapies [113, 164], the deleterious consequences of tumor therapy extend far beyond the cardiovascular all the way to the skeletal system [172, 177, 188]. Alterations in oxygen uptake, delivery, extraction, and utilization can occur from mouth to mitochondrion “ leading to a variation of clinical symptoms (Fig. 1)”. Molecular adaptations to cancer and cancer-related therapy impair CRF.

## Differential molecular regulation of cancer cells

Mitochondria are intracellular organelles which are involved in energy production through cleavage of phosphate from adenosine triphosphate and receive substrates from the cytoplasm to drive fatty acid oxidation, the tricarboxylic acid cycle, and the electron transport chain. They also contribute to synthesize amino acids, nucleotides, heme, lipids, NADPH, and modulate reactive oxygen species (ROS) for their own metabolic defense [223]. Mitochondrial chaperones, such as heat shock protein 60, assist unfolded proteins to reach their mature configuration [77]. Due to their central role in each cell`s energy production, mutations, be it cancer-induced or inherited, result in severe disturbance of cell function and culminate in systemic diseases, which especially affect the heart, nervous system, and muscle cell [45].

**Fig. 2:**
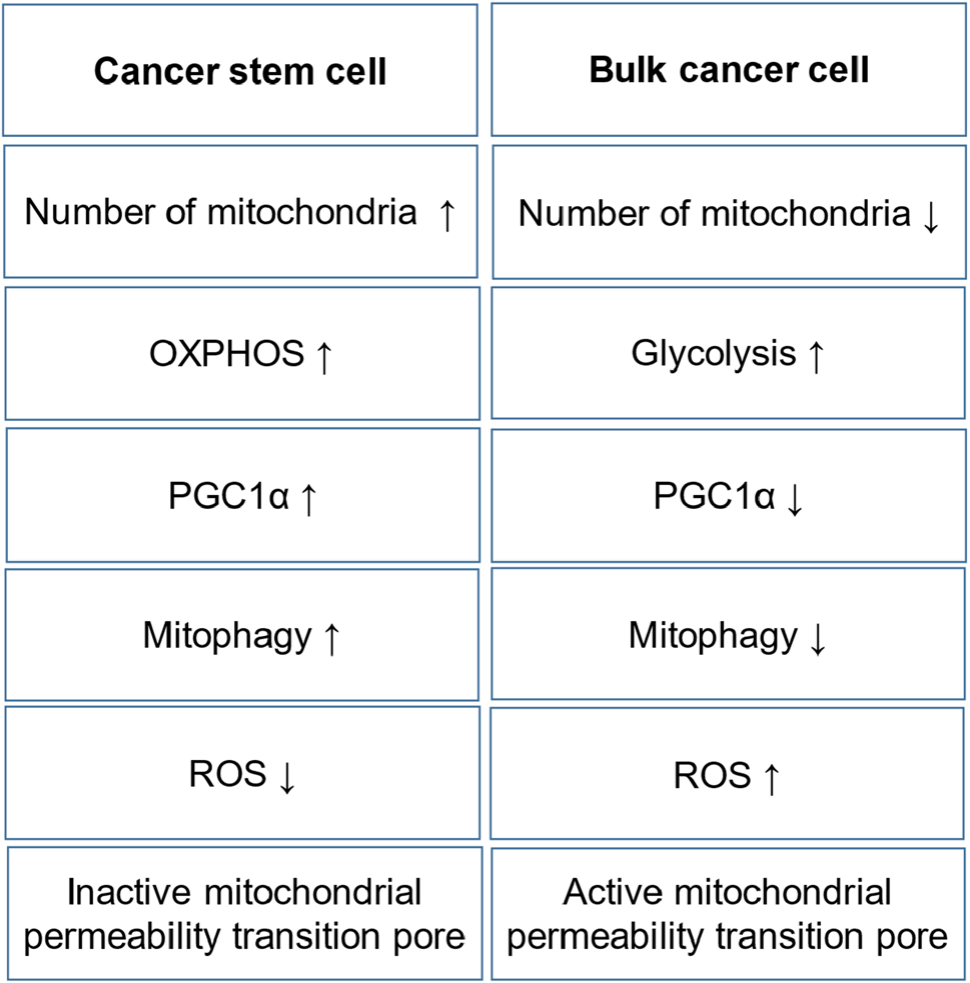
Differential molecular regulation of cancer cells. Cancer stem cells and bulk cancer cells display differential properties of intracellular communication. PGC-1α: Peroxisome proliferator-activated receptor gamma co-activator-1alpha. ROS: Reactive oxygen species

Cancerogenesis is a complex process, which can be induced by oncogenic driver mutations or loss-of-function of tumor suppressor genes [117]. It may also be linked to cell disturbances and affection of mitochondrial integrity through mitochondrial DNA mutations [55, 185], dysregulation of oxidative phosphorylation (OXPHOS) and ROS production [111], as well as impairment of mitochondrial repair and diversification through interference with mitochondrial fission, apoptosis, and mitophagy [91, 166]. The latter is a form of autophagy applied to eliminate non-functional components [119]. It is noteworthy that some tumor cells display a differential regulation of intracellular signaling, depending on whether they are fast or slowly dividing cancer cells. Intracellular metabolism may vary between cancer stem cells and bulk cancer cells [119] (for an overview see Fig. 2). For instance, many tumor cells display a high capacity of OXPHOS, while some mutations, such as Ras transformation, lead to a reduction of mitochondrial capacity [54].

Another mechanism of cancerogenesis and metastasis is imbalance of intercellular signaling and communication between mitochondria of different tissues, which is facilitated by mitokines, such as growth differentiation factor 15 and fibroblast growth factor 21 as well as humanin, a mitochondrial-derived peptide. Disturbances of mitokine signaling contribute to cancerogenesis and heart failure development and may be partly mitigated by regular exercise training [25].

Anticancer therapy usually attempts to inhibit specific pathways. Potential anticancer treatment is being investigated by interfering with mitochondrial ROS production [222], inhibition of autophagy [215], or enzymes of the tricarboxylic acid cycle [28]. Metformin, a complex I inhibitor, may show properties counteracting tumor growth in such tumors which specifically depend on OXPHOS, such as Ras-driven pancreatic cancer subtypes [120]. As tumor cells may partly display a differential regulation of cellular pathways (Fig. 2), inhibition of a single molecule or pathway may not cure cancer without negatively affecting healthy tissue. Pleiotropic effects of cancer treatment are needed to eliminate cancer and reduce side effects to a minimum. Anticancer treatment between 12 and 26 weeks can lead to significant decrease in CRF of up to 26% compared to a natural decline of 10% per decade of normal aging [48]. Cardiotoxic anthracycline therapy for instance increased the expression of the cellular senescence marker cyclin-dependent kinase inhibitor 2A which remained elevated up to one year in patients treated with primary breast cancer; this increase corresponds to almost 15 years of chronological aging [171]. By exerting a differential effect on cellular pathways, exercise may be a potential tool to stabilize CRF, antagonize tumor growth, improve intercellular signaling, and reduce systemic side effects.

## Molecular mechanisms of exercise

Growing evidence confirms the benefits of exercise on CRF in cancer patients [122]. Knowledge on molecular mechanisms of exercise in physiological skeletal and myocardial cells is increasing, but the molecular impact of exercise on cancer cells is insufficiently understood.

Anthracyclines, a common group of therapeutics in breast cancer, exert diverse effects on cancer cells including DNA damage and adduct formation, interferences with topoisomerase II, mitochondrial and cell membrane integrity, as well as ROS disturbances and initiation of apoptosis [32, 129]. These effects, however, are not only restricted to cancer cells, but also affect skeletal and myocardial muscle cells [129]. Exercise as a “pleiotropic drug” not only aims to reduce cardiovascular events but also strives to attenuate or prevent toxic effects of cancer therapy on the myocardium, skeletal muscle, and the endothelium. A few daily minutes of vigorous exercise for 3 days demonstrated a protective effect against cellular stress in post-menopausal women, in whom telomere length was longer compared to sedentary controls suggesting a protective effect against cell senescence and morbidity [161].

Effects of exercise displayed different results in animal cancer models: Chronic exercise demonstrated positive effects in a rat model before doxorubicin treatment and led to a better preservation of left ventricular function and greater cardiac expression of heat shock protein 72 [32], which increases cellular stress tolerance. A detrimental effect on tumorigenesis could be shown in a p53 deficient mouse training model [35]: Mice undergoing wheel running displayed a higher rate of mammary cancer than sedentary controls, despite positive effects of exercise on weight and fat content. Although no data are available on exercise intensity in these mice, these findings raised the question about a potential harm of exercise in patients prone to cancer development and a deficient mechanism to induce cell apoptosis. These findings were antagonized by a study taking serum from exercising patients with prostate cancer, in whom tumor apoptosis was induced by increasing cellular p53 content as compared to sedentary controls [115].

Exercise leads to sympathetic activation and release of catecholamines, such as epinephrine, which, if released excessively, may have a negative effect on the cardiovascular system. Exercise-induced, dosed release of epinephrine in five mouse tumor models led to the activation of cytotoxic natural killer cells and culminated in a 60% reduction of tumor growth [153].

Disturbances of cell–cell adhesion molecules, such as cadherins, can facilitate cancer metastasis [92]. Intestinal tumor growth was inhibited in two different mouse models undergoing voluntary wheel running leading to a reduction of the ratio of insulin-like growth factor 1 and its binding protein 3, while higher ratios were observed in colorectal cancers [88]. Nuclear beta-catenin, a main driver of intestinal tumorigenesis, was lowered and E-cadherin, an important cell adhesion molecule, was increased, which may be a mechanistic explanation to lower the rate of metastases [88].

Necrotic areas in bulk tumor cells may not be easily accessible to cancer therapy [129]. Aerobic training induces vascular wall shear stress and induces vascular endothelial growth factor release into the circulation, which triggers nitric oxide biosynthesis and results in vasodilatation and an increase of perfusion [123]. Mechanistically, an increase of perfusion, which is gained with exercise training, also leads to improved drug delivery to tumor cells [86].

Intracellular effects of AET have mainly been studied in myocardial and skeletal muscle cells [6, 123, 224]. Intracellular signaling is modified through AET by downregulation of protein kinase B, phospatidylinositol-3-kinase and the mammalian target of rapamycin, which are also targeted by chemotherapy [7].

Regular exercise increases mitochondrial function of skeletal and myocardial muscle cells by improving the activity of enzymes, such as cytochrome oxidase, succinate dehydrogenase, and succinate oxidase, and facilitates electron transport capacity to produce adenosine triphosphate [75]. Mitochondrial transcription is, among others, regulated by transcriptional coactivator peroxisome proliferator-activated receptor γ coactivator 1α (PGC-1α) [174]. Exercise induces PGC-1α dependent signaling pathways leading to the activation of calcium/calmodulin-dependent protein kinase [148], phosphorylation of AMP-activated protein kinase [81, 217], and activation of p38mitogen-activated protein kinase, which responds to ROS [81]. PGC-1α also interacts with nuclear respiratory factor 1/2, which facilitates activation of mitochondrial transcription factor A to activate mitochondrial proteins [60].

Exercise improves mitochondrial respiratory capacity, OXPHOS [160] and modulates ROS activity in skeletal, endothelial, and myocardial muscle cells [6, 76]. Importantly, exercise stimuli increase mitochondrial content independent of muscle fibre type [121]. Recruitment of type I fibres is best achieved with endurance training intensities below 40% of VO_2peak_, while type II fibres require more intense stimuli to optimize neuromuscular transmission [167]. Resistance training (RT) also enhances mitochondrial gene expression and mass [160]. Similar to low-intensity endurance training, high-intensity training (HIIT) triggers PGC-1α-dependent increase of mitochondrial synthesis, but achieves higher calcium release and can produce around 30% increase of mitochondrial content in a shorter period of time [123]. As early as six hours following exercise, mitophagy is enhanced by an increase of the adenosine mono-to-triphosphate ratio, which leads to activation of adenosine monophosphate- activated protein kinase and its downstream target Unc-51 like autophagy activating kinase 1. The mammalian target of rapamycin complex 1, a known suppressor of this activation, is inhibited [106].

**Fig. 3:**
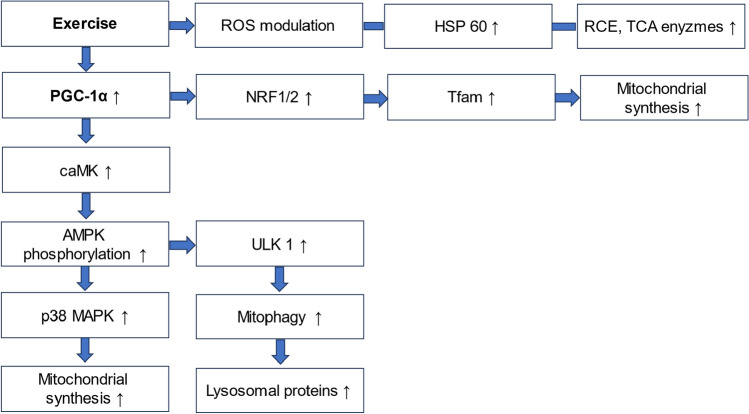
Selected exercise-induced molecular mechanisms in skeletal muscle cells. Exercise activates the central player (in bold) peroxisome proliferator-activated receptor gamma co-activator-1alpha (PGC-1α), which in turn facilitates the upregulation of calcium–calmodulin-dependent kinase (caMK) and 5`-adenomonophosphate-activated protein kinase (AMPK). Furthermore, p38 mitogen-activated protein kinase (p38 MAPK) is activated leading to an increase of mitochondrial synthesis. To stimulate autophagy of mitochondria and lysosomes, AMPK triggers activation of Unc-51 like autophagy activating kinase 1 (ULK 1). In addition, PGC-1α stimulates nuclear respiratory factor (NRF 1/2), which activates mitochondrial transcription factor A (tfam) and also results in enhanced mitochondrial synthesis. Furthermore, exercise facilitates modulation of reactive oxygen species (ROS), heat shock protein 60 (HSP 60), and upregulation of enzymes of the tricarboxylic acid cycle (TCA) and the respiratory chain (RCE)

Acute exercise leads to a localization of Parkin to skeletal muscle cell mitochondria as well as an increase in mitophagy flux, both processes which are coupled to PGC-1α [199]. Chronic exercise triggers activation of transcription factor EB as well as lysosomal markers mucolipin, Cathepsin D, and lysosomal associated membrane proteins 1 and 2 [95]. Thus, exercise-induced activation of lysosomal and autophagy-related genes facilitates cell organelles, such as mitochondria, to clear cell debris and uphold the necessary intracellular milieu to sustain performance (Fig. 3).

In summary, data on improvement of function of autophagy, ubiquitination, modulation of micro RNA, and regulation of calcium homeostasis are available in trained skeletal and myocardial muscle cells, while nitric oxide metabolism is positively influenced through exercise in endothelial cells [6]. Evidence from a Wistar rat model suggests that exercise also activates myocardial stem cells [203], which may bear potential for exercise regimens in patients suffering from cancer treatment-related cardiac dysfunction. Although it could be hypothesized that exercise may exert a differential, pleiotropic effect on cancer and physiological cells to preserve CRF, antagonize tumor growth, and reduce side effects of cancer treatment, there is insufficient data on which cancer- induced impairments of intracellular pathways can be positively modulated through exercise. This can only be achieved through translational studies analyzing both clinical endpoints and molecular mechanisms to resolve the inherent threat that exercise may not only protect physiological but also cancer cells [70].

## Cardiorespiratory fitness (CRF) and its assessment

The first documentation of a protective effect of swimming against cancer growth was demonstrated in a mouse model in 1952 [163]. While PA protected against cancer in humans and lowered cancer incidence by 48% and cancer-related mortality by 27% [198], cancer patients seem to be more sedative than adult controls and recommendations for PA are frequently (53–70%) not put into practice by cancer survivors [20]. Apparently healthy individuals and cardiooncological patients display a wide range of CRF, a predictor of cardiovascular morbidity and development of heart failure [94, 101, 145]. An improvement of 1 metabolic equivalent (MET, which is 3.5 ml/kg/min) of exercise performance has been associated with a 10–25% relative risk reduction in overall mortality [90], while low CRF has been associated with higher morbidity, poor quality of life, reduced cardiac performance during exercise and a worse cardiovascular risk profile in cancer patients [44]. In a single-centre cohort analysis of 1632 patients (58% male; 64 ± 12 years) with adult-onset cancer, treadmill testing was performed at a median of 7 years after primary cancer diagnosis. The adjusted risk of all-cause, cardiovascular, and cancer mortality decreased by 26%, 14%, and 25% with each one MET (3.5mlO_2_/kg/min) increment in CRF [61]. CRF decreases between 5 and 26% during exposure to different systemic treatment regimens [80, 84] and does not entirely recover following cessation of treatment [3, 85]. Currently, no reimbursement of structured exercise training as a part of the rehabilitation process of cancer patients has been established, despite its proven benefit on CRF improvement of cancer patients [103, 107].

CPET is the gold standard to assess CRF [62]. Evidence in a cancer population is mainly limited to pre-operative risk stratification, especially in lung [64], colon [213], and rectal [214] cancer. Whether CPET can predict cardiovascular events if it is performed prior to cardiotoxic cancer treatment is unknown. Current guidelines recommend CPET as to be considered in cancer patients with exertional limitations, especially those treated with higher doses of anthracyclines and/or radiotherapy including the heart [122]. CPET is considered useful if high cardiovascular toxicity is to be expected at baseline as well as in patients who develop chemotherapy-related cardiac disease during cancer treatment [122]. CPET is suggested if left ventricular function declines during or after therapy [57] and if subjective exercise limitations need to be differentiated into cardiac vs. non-cardiac origin, also in combination with stress echocardiography [183]. CPET could also be used to detect early deterioration of CRF in patients under immune-checkpoint inhibitors in addition to repetitive troponin measurements to better risk stratify cancer patients [201].

Using CPET a holistic and integrative approach could be taken in cancer patients to early diagnose and adequately train this vulnerable group [112, 157]. As many cancer patients display preserved ejection fraction, guidance could be retrieved from the position paper of exercise testing in heart failure with preserved ejection fraction [63]. Ejection fraction as such has been shown to be a modest marker to express CRF, and maximal as well as submaximal CPET variables may add benefits to guide therapy and follow-up visits [204, 207, 209, 212]. A depiction of useful CPET variables, most of which still have to be validated in a cancer cohort, is listed in Table 1.

**Table 1:** Maximal and submaximal variables of cardiopulmonary exercise testing

Submaximal variables	Maximal variables
OUES	VO_2peak_
VO_2_ at VT1	Circulatory power
COP	Ventilatory power
VE/VCO_2_	Peak O_2_ pulse
VD/VT	VO_2_/W trajectory
EOV	Increase of PETCO_2_
Plateauing of O_2_ pulse	Peak heart rate
	HRR
	HRR1
	Alveolo-arterial oxygen difference

*CP* circulatory power, the product of peak systolic blood pressure and peak oxygen consumption. *COP* cardiopulmonary optimal point; it represents the nadir of the oxygen equivalent. *EOV* exercise oscillatory ventilation. *OUES* oxygen uptake efficiency slope, the logarithmic relationship between oxygen uptake and minute ventilation. *HRR* heart rate reserve, difference between peak and resting heart rate. *HRR1* heart rate recovery one minute after exercise termination. *PETCO*_*2*_ Endtidal carbon dioxide. *VO*_*2*_* at VT1*: oxygen uptake at the first ventilatory threshold. *VO*_*2peak*_: peak oxygen uptake. *VD/VT*: dead space ventilation. *VP*: ventilatory power, the ratio of peak systolic blood pressure and VE/VCO_2_. *VO*_*2*_*/W* oxygen consumption per Watt of achieved power

## Components of physical fitness and exercise

**Fig. 4:**
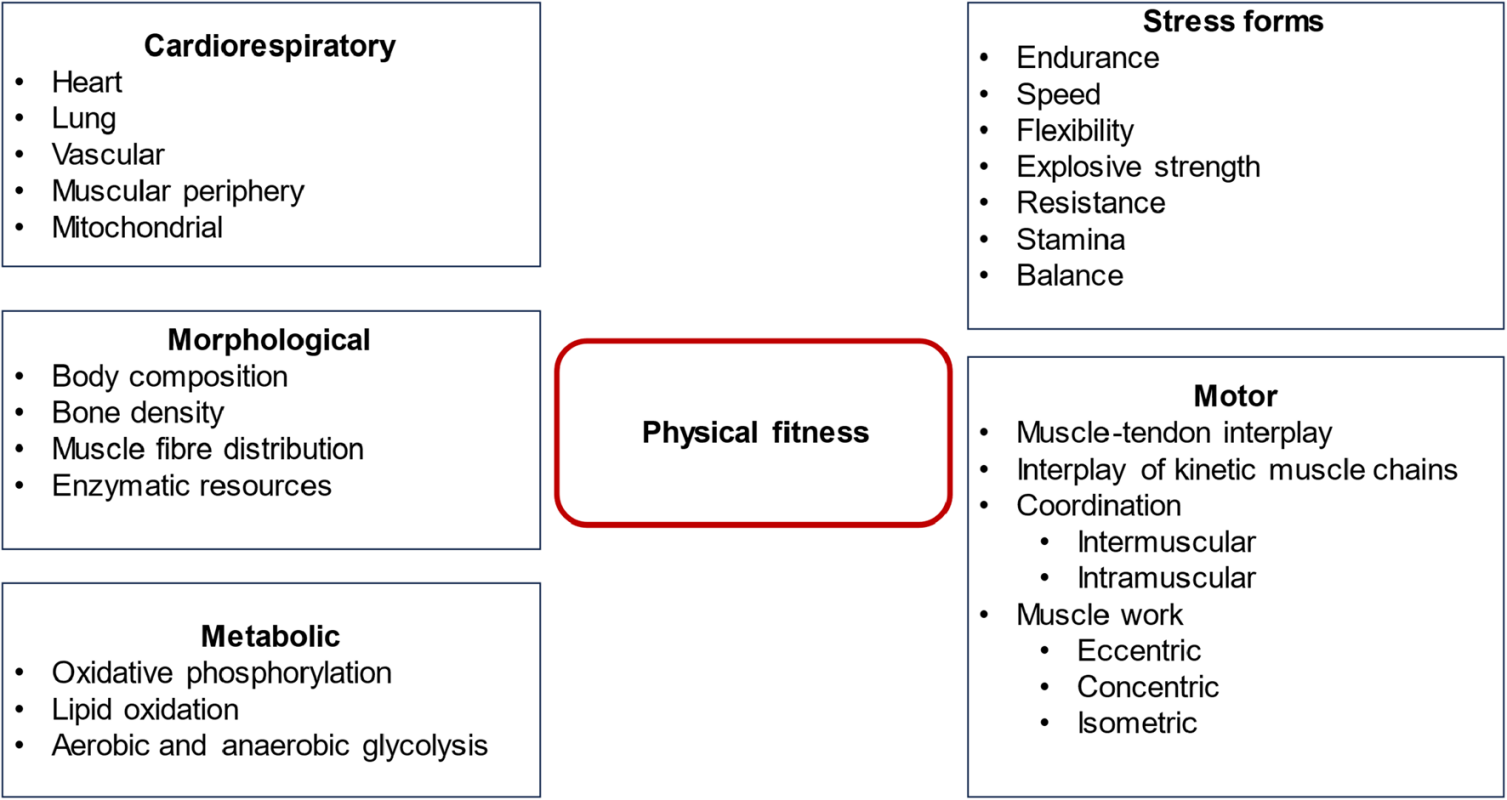
Components of physical fitness. Physical fitness is a state of multiple components, only one of which is cardiac function

Sports cardiology recommendations propose a weekly “dose” of PA (which depicts unstructured activity as opposed to structured exercise training) of at least 150 min of moderate intensity, preferably on most days of the week [154]. This recommendation can be applied to cancer patients, since a meta-analysis on 50.000 breast and colon cancer patients has found a reduction of total mortality risk of 24% and 28% in active cancer survivors exercising for 150 min per week compared to sedentary controls [176]. Benefits of exercise training are not limited to improvement of cardiac function, but have beneficial effects on metabolic, muscular, motor, and morphological aspects of the human organism (see Fig. 4) [154]. Exercise training can also be performed in severely compromised patients if expertise in exercise sciences and training are available [206, 208].

Guidelines of sports cardiology also recommend variations in exercise frequency (sessions/week and bouts of exercise), intensity, duration (per day or week), or type (resistance, endurance, speed, flexibility, coordination) [154].

Endurance training recommendations are derived from % of VO_2peak_, % of peak heart rate, or % of heart rate reserve (the difference of resting and peak heart rate) [154]. Using % of VO_2peak_ as a determinant to recommend endurance exercise prescription requires full metabolic exertion on initial CPET testing (at least requiring a respiratory exchange ratio, RER, > 1.05), which may be hard to achieve in severely compromised patients, such as those exercising during chemotherapy or advanced heart failure [41]. Comparison of VO_2peak_ is limited between treadmill and bicycle testing because efficiency of oxygen consumption, among others, depends on applied muscle mass and body composition, with deviations of up to 20% [124, 150]. Current exercise physiology units and software specifications often apply the validated Wasserman-Hansen equation for reference values of VO_2peak_ [66], but recent data suggest that adapted reference values gained from the FRIEND registry may be superior to anticipate morbidity [31, 144, 155].

Determination of exercise corridors from heart rate has limitations because individual heart rate performance curves or the application of beta- blockers complicate adequate prescriptions [74, 219]. An easy and practical approach to steer endurance exercise intensity is the BORG scale [21], which depicted physiological measures in a large young male and female Caucasian cohort (*n* = 2560, median age 28, IQR 17–44 years) [175]. Standard corridors for endurance training are shown in Table 2 [154]:

**Table 2:** Corridors for endurance training. adapted according to [154]

Intensity	VO_2peak_ [%]	HR_peak_ [%]	HRR [%]	RPE	Training zone
Low	< 40	< 55	< 40	10–11	Aerobic/alactacid
Moderate	40–69	55–74	40–69	12–13	Aerobic/alactacid
High	70–85	75–90	70–85	14–16	Aerobic/lactacid
Very high	> 85	> 90	> 85	17–19	Anaerobic/lactacid

*HRR* Heart rate reserve, difference between resting and peak heart rate. *HR*_*peak*_ Peak heart rate. *RPE*: Rate of perceived exertion, adapted from the BORG scale ranging from 6 (really easy) to 20 (maximal effort) points. *VO*_*2peak*_ Peak oxygen consumption

Intensity of RT is commonly determined with the one repetition maximum (1RPM). Moderate intensity between 30 and 50% of 1RPM with 15–30 repetitions is considered to be muscular endurance training [154]. In cardiac patients eight to ten resistance exercises are still recommended to cover most large muscular groups [154, 180] with resting intervals of 3-5 minutes [156]. In cancer patients there is limited data on comparisons between types of RT, such as dynamic vs. static/isometric exercise, which may create different muscular responses and differential effects on long-term blood pressure [154]. A meta-analysis of eleven randomized trials on cancer survivors exercising during and after chemotherapy showed that upper limb strength seems to improve slightly better at low-to-moderate intensities below 75% of 1RPM (*p* = 0.042) [186].

## Clinical effects of exercise training in cancer patients

The effects of exercise on cardiovascular disease, heart failure, mortality, and surrogate variables have primarily been studied in breast cancer patients (see Appendix). The Framingham risk score (FRS) was higher in overweight early- stage breast cancer patients [15, 58]. Following AET and RT, FRS was significantly reduced in the intervention group corresponding to an 11% (CI: − 15 to − 5%) reduction on the FRS-predicted 10-year risk of developing cardiovascular disease [110].

The association between PA and cardiovascular disease was investigated in a prospective study with non-metastasized breast cancer patients (*n* = 2973, mean age 57 years, median follow-up of 8.6 years): Exercise was measured by MET-h/week and patients were classified into three groups: (1) 2–10.9, 11–24.5, and 24.5 MET-h/week. Cardiovascular events decreased across increasing total MET-h/wk categories (*p* < 0.001) in multivariable analysis. Compared with < 2 MET-h/week, the adjusted HR was 0.91 (CI: 0.76 to 1.09) for 2 to 10.9 MET-h/week, 0.79 (CI: 0.66 to 0.96) for 11 to 24.5 MET-h/week, and 0.65 (CI: 0.53 to 0.80) for ≥ 24.5 MET-h/week. A similar trend was observed for the incidence of coronary artery disease and heart failure (*p* values < 0.05). Importantly, further analysis revealed that adherence to national exercise guidelines for adult patients with cancer (≥ 9 MET-h/week) was associated with an adjusted 23% reduction in the risk of cardiovascular events in comparison to < 9 MET-h/week (*p* < 0.001; Appendix) [87]. Similarly, the large Women`s Health Initiative (WHI) study on non-metastatic breast cancer patients (*n* = 4015) with a long-term follow-up (median 12.7 years) also demonstrated a reduction of cardiovascular events with higher self-reported exercise levels prior or at the time of cancer diagnosis: Comparing ≥ 9MET-h/week (*n* = 1976) vs. < 9 MET-h/week (*n* = 2039), HR was 0.77 (CI: 0.62 to 0.95) for age-adjusted cardiovascular events and 0.56 (CI: 0.35 to 0.89) for coronary heart disease associated death [149, 151].

In breast cancer patients, trials demonstrated benefits of training in chemotherapy naïve patients [146], during neoadjuvant chemotherapy [86], during [37, 78] and after adjuvant chemotherapy [52, 67, 69, 87, 110], and during maintenance therapy on aromatase inhibitors [82] (see Appendix). Typical symptoms during radiotherapy, such as fatigue or arthralgia, were reduced through AET [118] and tumor relapse may be reduced in physically active individuals [7]. A meta-analysis of exercise in breast cancer patients included 22 prospective cohort studies (follow- up of 4.3 to 12.7 years, 123 574 participants, 6898 all-cause deaths, 5462 breast cancer-related deaths or recurrences) and showed fewer events for tumor progression (HR = 0.72, CI: 0.56–0.91) and tumor relapse (HR = 0.79, CI: 0.63–0.98) if women exercised before and after the diagnosis [105]. Patients reporting higher PA on tumor diagnosis demonstrated lower risk of all-cause (HR = 0.82, CI: 0.70–0.96) and breast cancer-related death (HR = 0.73, CI: 0.54–0.98) than more sedentary controls. However, this large meta-analysis also highlighted the heterogeneity of studies and variation of defining and assessing PA and AET [105].

Outcome data on exercise trials in urogenital cancer is scarce, with exercise trials showing a positive effect on CRF [5] and adverse health outcomes [53] in testicular cancer survivors. Similarly, CRF was improved [36] and tumor progression was reduced [165] in exercising prostate cancer patients (for details see Appendix).

Data on cardiovascular outcome or overall mortality reduction in colon cancer exercise trials are not yet available, but AET reduced post-operative complications [197], improved disease-free survival [131], quality of life [22], and decreased circulating markers associated with the formation of micro-metastasis, such as soluable intercellular adhesion molecule-1 [152] (for details see Appendix).

Exercise trials in lung cancer patients following surgery is scarce and no studies are available on benefits in overall survival due to exercise interventions. However, a meta-analysis of lung cancer exercise trials demonstrated that pre-operative exercise-based training can improve vital capacity and forced expiratory volume (standardized mean difference: 0.38 l, CI: 0.14–0.63 l and 0.27 l, CI: 0.11–0.42 l) before surgery and reduces in-hospital length of stay (mean difference in hospital days: − 4.83 days, CI: − 5.9–3.76 days) as well as post-operative complications (risk ratio: 0.45, CI: 0.28–0.74) after lung resection surgery [179]. A Cochrane meta-analysis of randomized controlled trials of participants with non-small cell lung cancer analyzed patients who had undergone lung resection [29]: Patients were allocated to receive either AET, RT, or a combination of both. Compared to usual care (UC), VO_2peak_ (mean difference 2.97 ml/kg/min, CI: 1.93–4.02 ml/kg/min) and 6-min walk distance (6MWD, mean difference: 57 m, CI 34–80 m) were greater in the intervention group. In addition, improved force-generating capacity of the quadriceps muscle (mean difference 0.75, CI 0.4–1.1) was displayed [29].

Data on exercise interventions in head and neck cancer (HNC) patients are scare [26], but reduced loss of muscle mass [170] and improvement of functional capacity has been reported in HNC patients [8, 168, 169] (see Appendix).

Randomized trials on exercise-based reduction of cardiovascular events or mortality are not available in hematological cancers. No improvement of 6MWD was achieved in an exercise trial in patients with acute leukemia [23], while exercise intervention was superior to UC in surrogate markers such as quality of life and CRF in patients with Hodgkin and non-Hodgkin lymphoma [38] (see Appendix).

## Limitations, recommendations and future directions- a critical appraisal

Current guidelines on cardiooncology acknowledge the role of cardiac rehabilitation and AET as a potent multi-targeted therapy to prevent and treat competing mechanisms of chemotherapy-related cardiovascular toxicity (CTR-CVT) [122]. Exercise is recommended to improve CRF in cancer patients [178], and to reduce the burden of cardiovascular injury and traditional cardiovascular risk factors [177]. To ensure optimized response to exercise, adaptation of basic principles of exercise sciences to improve CRF should be transferred to cancer patients [173]. In cancer patients several studies were performed in the form of home-based training, although the benefits of supervised training have been demonstrated [200]. CPET-based supervised training may best account for the individual exercise needs of this vulnerable group of patients.

CPET may not only be performed at baseline, but also could be repeated throughout the treatment process to re-evaluate CRF to detect early circulatory limitations, for instance decline or plateauing of O_2_ pulse as a surrogate for reduced stroke volume and peripheral oxygen extraction [210]. This may even necessitate the application of different CPET reference values for cancer patients [132]. Reduced CRF should not be merely separated into cardiac and non-cardiac origin, but one also needs to pay attention to reduced oxygen extraction, which may be a major limiting factor in cancer patients despite preserved ejection fraction [104]. It has been shown that in elderly patients with heart failure and preserved ejection fraction, VO_2peak_ was better associated with arteriovenous oxygen extraction than cardiac output, highlighting the importance to analyze peripheral muscular response to exercise [68]; a phenomenon which can also be observed in cancer patients. Chemotherapy- induced loss of hemoglobin needs to be compensated by enhanced peripheral small-muscle blood flow and O_2_ extraction, which, however, is insufficient to maintain whole body performance [99]. Increased myosteatosis during chemotherapy suggests hampered fat oxidation [99]. In clinical practice, focusing on early (even before initiation of chemotherapy) aerobic-alactacid training may antagonize this process.

As VO_2peak_ is dependent on metabolic exertion, non-exertional CPET variables, such as oxygen uptake efficiency slope [14], minute ventilation to carbon dioxide production, exercise oscillatory ventilation, and the cardiorespiratory optimal point may be of additional benefit in cancer patients, as this might prevent potential adverse events at peak exertion. Transition from aerobic to anaerobic metabolism can be illustrated by non-exertional variables, which may also be useful to monitor metabolic changes during cancer treatment. Combining right heart catheterization and CPET may complement the image to meticulously display exercise limitations and guide exercise training and drug therapy [205, 211].

Moderate continuous training (MCT), which is usually performed at 50–75% of peak heart rate, is still the backbone of AET. As opposed to MCT, HIIT uses repetitive bouts of high-intensity exercise intertwined by a variable duration of active recovery phases. The „Norwegian protocol “ originally used 4 x 4 min intervals at 90 and 95% of peak heart rate and included 3 min cool-down phases between 50 and 70% of peak heart rate using uphill treadmill walking [218]. HIIT improved VO_2peak_, endothelial and mitochondrial function as well as capillary density in the skeletal muscle [59, 123, 181]. A meta-analysis demonstrated higher efficacy to improve VO_2peak_ in apparently healthy individuals [140], while a meta-analysis of heart failure trials showed a trend toward higher VO_2peak_ improvement in HFrEF and even more so in HFpEF [59]. It has to be taken into account that vulnerable groups, such as HFrEF and cancer patients, may fail to sustain intensity corridors of the classical HIIT protocol [41], which may require adapted HIIT protocols with shorter bouts of high-intensity exercise and longer periods of recovery to avoid rapid lactate accumulation. Such adapted HIIT protocols are safe and effective in advanced heart failure patients with LVAD [9, 142, 208] and may prove useful in cancer patients.

In cancer patients supervised AET (including MCT and HIIT) is safe, well tolerated, and capable of improving VO_2peak_ as well as attenuating CTR-CVT risk [202] and cardiovascular risk factors [110, 184]. A recent meta-analysis of controlled trials in cancer patients and survivors using post-intervention CPET did not find differences of VO_2peak_ in MCT and HIIT (mean difference = 2.03 ml/kg/min, CI − 0.75–4.83 ml/kg/min) [202]. This conveys a slightly different effect compared to heart failure trials, which have shown a trend to more effective HIIT [59, 181]. This might be explained by the heterogeneity of exercise studies in cancer patients. We believe that it is essential to conduct studies using adapted HIIT in different cancer entities and states of disease (pre-, during-, and post-treatment) to increase the portfolio of available exercise programs. The need to establish more tailored exercise programs in cancer patients has been acknowledged by American [57] and European [122] recommendations. It also has to be highlighted that not only improvement of CRF should be achieved but also attenuation of chemotherapeutic side-effects, such as reduced quality of life and fatigue; HIIT is able to improve and maintain these effects for months following exercise intervention [4, 139]. Whether HIIT is also feasible in frail and older cancer patients still needs to be shown [126].

Performing AET during radiochemotherapy may need to be designed in a nonlinear periodization model to account for fluctuations of symptoms through therapeutic cycles without compromising the benefit of training [50, 52]. The Brexit trial is the first and only randomized controlled trial with a sufficient follow-up period to assess prevalence of functional disability (defined as VO_2peak_ ≤ 18 ml/kg/min) after 1 year in an exercise training group compared to UC in breast cancer patients within two weeks of induction of chemotherapy (*n* = 52 per group, mean age 50.3 vs. 51.2 years). It made use of a combination of AET (MCT and HIIT) and RT. Importantly, both intensity and duration were progressively increased during macrocycles of exercise and were reduced by 5% in the week following anthracycline-based chemotherapy. Intensity of AET was based on % of heart rate reserve at the first ventilatory threshold (VT1) up to 85–95% of peak heart rate [52]. As no beta-blockers were prescribed in both groups, this approach to guide training intensity with heart rate monitoring (wrist watch) can be considered accurate. The Brexit trial used a supervised training protocol (three times/week for 30–60 min) for the first 12 weeks. AET was performed as MCT (20–30 beats/minute below %HRR at VT1) and HIIT, the later consisting of 4 x 2-4 minutes of interval training of % HRR at VT1 up to 95% of peak heart rate. It may be interesting to compare the efficacy of this approach with shorter bouts of HIIT. Importantly, the Brexit trial also implemented RT of various intensities between 60 and 70% (weeks 1–6) and 70–85% of 1RPM (following week 7) by using eight guided exercises of upper and lower muscle groups. It may be reasonable to modify these exercises with multi-component movements to recruit adjacent muscle chains and further improve intermuscular coordination. To study the response of the cardiovascular system around chemotherapy in more detail, it may be useful to perform CPET follow-up and lactate measurements during this critical period to delineate exercise limitations of cardiac output and peripheral oxygen extraction. This can be analyzed by CPET variables, such as flattening of peak O_2_pulse and VO_2_/W (Table 1), which have already shown prognostic [159] and pathophysiological impetus [210] in heart failure cohorts. Such variables should be analyzed and validated in cancer patients to survey response to chemotherapy. In addition, compound variables such as ventilatory [49] and circulatory power [34] could have the potential to better display CRF than VO_2peak_ alone.

### Limitations of exercise trials in sports cardio-oncology

Exercise trials are heterogeneous (see Appendix) because many aspects must be considered and sport scientific expertise is necessary to address the metabolic needs of vulnerable populations, such as cancer and/or heart failure patients: Steering RT intensities based on 1 RPM is reasonable to objectively measure intensity. However, in vulnerable populations, such as patients with cancer, advanced heart failure or left ventricular assist devices (LVAD) as well as exercise-naive patients, performing 1 RPM may not always be feasible. These patients often do not have the coordinative capabilities to perform such tests. In such cases a stepwise increase of RT intensity during the training cycle could be applied, with the main focus on building up smooth coordination of kinetic muscle chains. Our group has acknowledged this approach in an exercise training study in LVAD patients [208], which could also be adapted in a cancer population.

In the literature, no clear recommendations are provided for a major determinant of exercise limitation: Exercise density, which is the relationship between intensity and duration and the intertwined active or passive recovery periods. From a physiological point of view this aspect is of utmost importance when higher intensities are applied and local lactate clearance is a limiting factor, such as in heart failure and/or cancer patients. Studies with different exercise protocols on HIIT have been elaborated paying tribute to this methodological issue [9, 123, 208]. A large multi-center randomized trial in advanced heart failure patients showed that almost half of the patients did not adhere to the scheduled training corridors of HIIT [41], which may be due to limited availability of lactate measurements during training and may have been caused by early lactate accumulation and associated premature reduction of training intensity.

No uniform consensus exists on the determination of training corridors, while usually % of peak heart rate or heart rate reserve are used to separate MCT from HIIT. However, the heart rate performance curve varies considerably among individuals [74] and can be influenced by beta-blockers [219], which are often used in cancer patients with cardiovascular comorbidities; percentages of VO_2peak_ or peak workload may be preferrable substitutes to guide training [208, 219]. It is noteworthy that great care should be taken in the interpretation of exercise studies, because terminology of prescribing exercise intensities based on ventilatory and lactate thresholds varies [19] (see Appendix). Comparability between MCT and HIIT is also hampered by the lack of isocaloric exercise stimuli, which is a prerequisite if effects on CRF are analyzed [59, 181]. Further studies comparing MCT and HIIT in cancer need to take this into account.

**Fig. 5:**
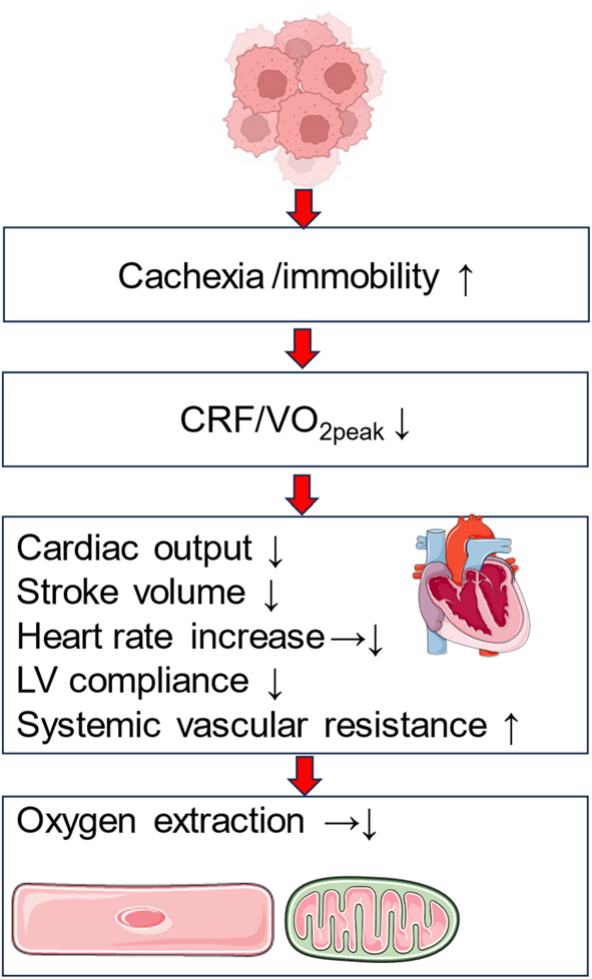
Exercise limitations of cancer patients. Cancer patients display reduced cardiorespiratory fitness (CRF) expressed through reduced peak oxygen consumption (VO_2peak_), which is often facilitated by cachexia and/or cancer-induced immobility. VO_2peak_ reduction in cancer patients is a composite of lower cardiac output, stroke volume, heart rate increase, and peripheral oxygen extraction during exercise. Reduced left ventricular (LV) compliance and increased systemic vascular resistance also contribute to VO_2peak_ reduction. Created with BioRender.com

Data from randomized, controlled, supervised exercise interventions in cancer patients are available primarily in younger, non-cachectic individuals and do not include frail and sarcopenic patients [83, 130]. Sarcopenia and cachexia are a prominent complication in cancer patients and further promote CRF reduction (Fig. 5) [16]. Early recognition of sarcopenia and compilation of nutritional and exercise pathways are important goals in treating cancer patients [12, 46] and need to be addressed in future trials of sport cardio-oncology.

### Recommendations for exercise prescription in cancer patients

We believe that there cannot be a specific exercise program for each cancer entity, since exercise responses are expected to be different in each individual, also depending on (heart failure) co-medication, which may become necessary in the presence of cancer treatment-related cardiac dysfunction. As drug therapy may change before, during, and after chemotherapy we suggest repetitive CPET to achieve adequate metabolic corridors to sustain or even improve CRF. As there are many caveats of deriving exercise corridors from exertional variables, such as VO_2peak_ [124] and peak heart rate [74], we suggest determination of AET exercise corridors from VT1 and the second ventilatory threshold (VT2). It should be noted that reduced VO_2peak_ in cancer patients is the result of several mechanisms, including reduced cardiac output [51], reduced stroke volume increase [17], reduced left ventricular compliance [17, 83], lower heart rate response, higher systemic vascular resistance [18], impaired peripheral oxygen extraction [138], cachexia and immobility [10, 130]. Drug-related symptoms, such as fatigue or polyneuropathy, may also reduce adherence to exercise training and may ultimately reduce VO_2peak_ during chemotherapy [96, 98]. An elegant study addressed this issue and performed 30 min of vigorous treadmill walking 24 h prior to every anthracycline therapy [97, 100], no adverse events and 100% adherence to exercise were achieved. Adherence to AET and symptoms refraining patients from participation in exercise sessions always need to be taken into account whenever effects of exercise interventions on VO_2peak_ and other CPET variables are interpreted in cancer patients. A summary of cancer-induced exercise limitations is provided in Fig. 5:

HRR, the difference between resting and peak heart rate, which is often used in exercise trials, also has limitations: (1) Application of beta-blockers impacts on HRR, and (2) Taking resting heart rate prior to exercise testing may not represent “true” resting heart rate due to enhanced sympathetic tone in anticipation of testing. Thus, “true” resting heart rate may need to be recorded repetitively directly after waking up. This, however, is not a standard in trials of sports cardio-oncology. Implementation of a home-based monitoring in the form of wearables or apps should help to better assess “true” resting heart rate and have proven their benefit to raise awareness for physical activity in breast cancer patients [33, 42, 127]. An “app on prescription” is a promising new approach to increase the patient’s compliance to exercise and also aids to detect deterioration of health status and could be used in cancer similar to heart failure patients [1]. A combination of wearables and artificial intelligence-based approaches can be regarded promising to increase cancer detection [40, 108], treatment [221], as well as an improvement of lifestyle interventions [30] and early recognition of cardiac disease and heart failure [13, 56].

We suggest establishing exercise corridors above VT1: Moving beyond VT1, which is the first significant increase of ventilatory effort, can be considered the minimal stimulus to accomplish a training effect and should also be achieved in frail cancer patients. HIIT should be applied by short bouts close to VT2. Adaptations of intensity should be made during chemotherapy and CPET should be performed before, during, and after chemotherapy, as well as following adaptations of heart failure therapy. We also suggest combining MCT and HIIT, since both are safe and improve CRF.

**Fig. 6:**
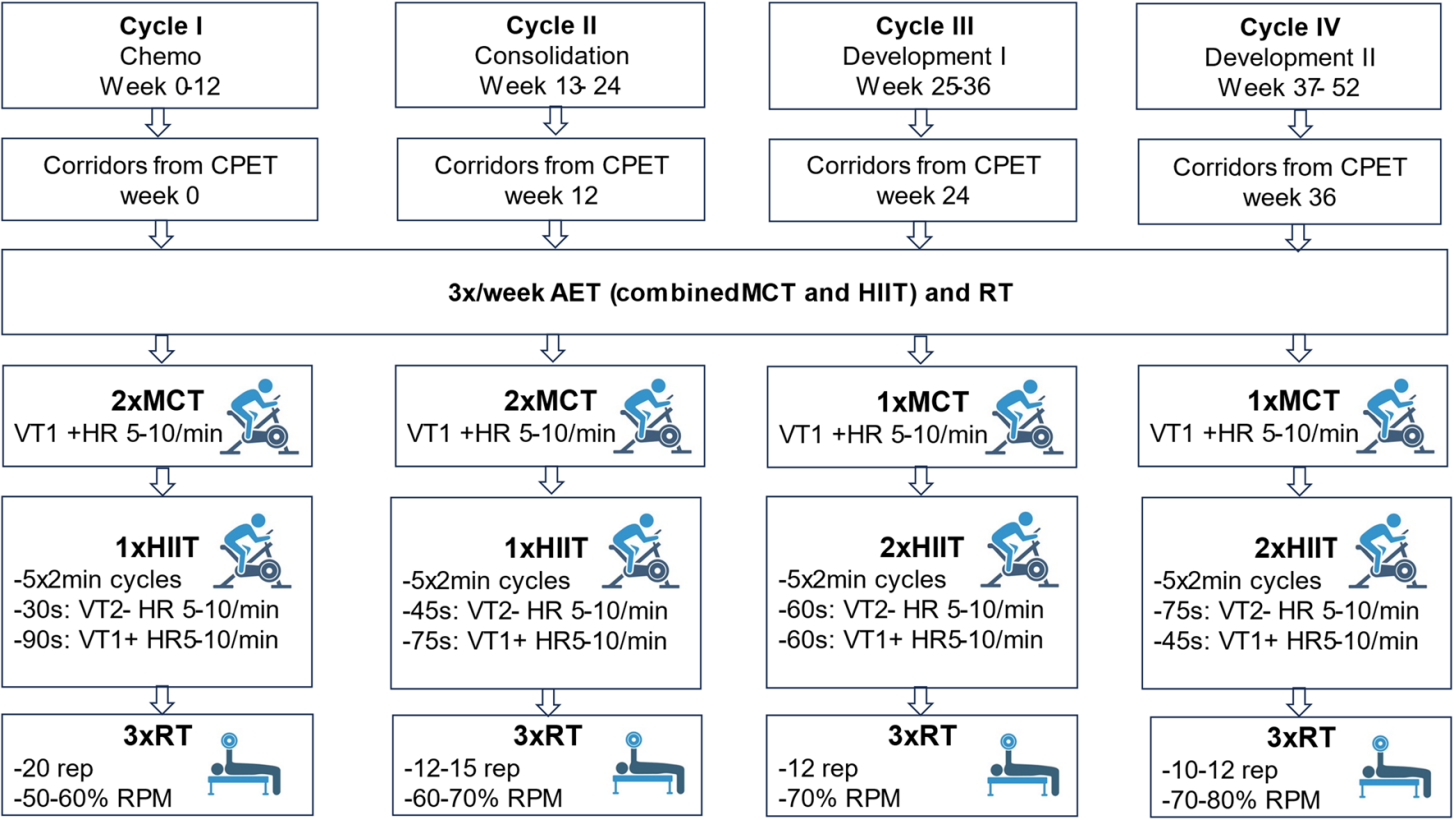
An example for a tailored exercise prescription in a breast cancer patient undergoing 12 weeks of chemotherapy and a 12-month- follow-up is provided. Exercise is performed 3x/week (30 min each) including aerobic exercise training (AET) consisting of both moderate continuous (MCT) and high-intensity exercise training (HIIT) and additional resistance training (RT). A year of training is separated into four cycles of 12 weeks each, with the first cycle integrating chemotherapy. Before each cycle cardiopulmonary exercise testing (CPET) is performed to adapt training corridors of AET. MCT is performed at 5–10 beats/min (HR, heart rate) above the first ventilatory threshold (VT1), while HIIT consists of five intense cycles with 2 min each using short bouts just below (5–10 beats/minute) the second ventilatory threshold (VT2). RT is increased in intensity over the year starting with more repetitions (rep) and lower intensities (derived from the one repetition maximum, 1 RPM). Frequency of MCT and HIIT is varied across the cycles, RT is performed 3x/week. Created with BioRender.com

An example of a tailored exercise periodization model for a breast cancer patient during and after chemotherapy is shown in Fig. 6:

HIIT and MCT have beneficial effects on mitochondrial function and cell–cell communication, which both improve cellular function [24, 123]. In turn, these effects as efforts to protect the heart and cardiovascular system could possibly also protect cancer cells and remain a matter of concern [70–72]. Consequently, the differential effect of AET on preservation of CRF and simultaneously antagonizing cancer growth, metastasis, and metabolism needs to be further demonstrated by translational trials. These trials in sports cardio-oncology should incorporate clinical outcome measures, underlying microscopic mechanisms, and circulating biomarkers which may help in earlier detection of cancer therapeutics-related cardiac dysfunction [189].

Cardiovascular exercise response in cancer patients has primarily been analyzed with stress echocardiography and magnetic resonance imaging [83], although the gold standard to assess hemodynamics is right heart catheterization. There is a strong need to validate current results derived from imaging with simultaneous CPET and exercise right heart catheterization.

In summary, there is a strong need to create more homogeneity in cancer exercise trials, which can only be achieved by objective measurement of baseline performance, and more precise training surveillance [27, 125, 128]. This task can only be achieved through close interaction of physicians and exercise physiologists.

## Conclusion

Aerobic exercise training is recommended as a part of adjuvant and neoadjuvant therapy as well as during chemotherapy [177, 220]. Unfortunately, randomized controlled trials using CPET prior to exercise initiation to adequately steer training intensity and improve cardiovascular and overall outcome are scarce in the cancer population. There is insufficient evidence on the most suitable type (supervised vs. home-based), content (endurance, resistance, or combined), intensity (moderate continuous vs. high-intensity interval), or density (short vs. longer bouts of stimuli with active vs. passive recovery periods) of training in cancer patients. Conducting randomized controlled exercise trials in patients undergoing radiochemotherapy are demanding and may be subject to significant drop-out rates during longer exercise intervention periods. Valid clinical endpoints, such as cardiovascular and overall mortality, are hard to achieve in younger cancer populations, who require a long follow-up period. Implementation of wearable devices into home-based exercise training and offers of public health providers (e.g., cancer sports groups) may improve adherence to AET and RT in cancer survivors.

## Supplementary Information

Below is the link to the electronic supplementary material.

Supplementary file1 (DOCX 73 KB)

## Data Availability

There was no collection of original data in this review. All information regarding this review will made available by the corresponding author on reasonable request.
