# Supplementary material for: Exercise, cancer, and the cardiovascular system: clinical effects and mechanistic insights
Source: Basic Res Cardiol. 2024 Feb 14;120(1):35–55. doi: 10.1007/s00395-024-01034-4 (PMC11790717; doi:10.1007/s00395-024-01034-4)
Supplement: Supplementary file 1 — Supplementary file1 (DOCX 73 KB) [file 395_2024_1034_MOESM1_ESM.docx]

**Appendix**

| **Trial** | **Methodology** | **Type of cancer** | **Sample size** | **Phase of treatment** | **Comorbidities** | **Exercise intervention** | **Outcome** |
| --- | --- | --- | --- | --- | --- | --- | --- |
| Lee et al. 2019 [110] | Single- center, prospective randomized | Breast | n=100 | - < 6 months post-treatment  - Stage I to stage III | Sedentary | AET (65%-80% HR_max_) and RT.  3x/week supervised for 16 weeks.  (≥150min AET, 2-3x RT/week | FRS was lower in AET (mean reduction: −9.5, CI: −13.0 to −6.0).  FRS predicted 10-year CVD risk (mean reduction: −11.0; CI, −15.0 to −5.0).  Reduction in FRS was maintained during the 3-month follow-up in AET compared to baseline (mean, −10%, CI, −15.5 to −4.2) |
| Jones et al. 2016 [87] | Prospective, two registry-based, regional cohort studies | Breast | n=2973 | - Nonmetastatic  - stage I-IIIA  - Completed adjuvant therapy  - 82% ER positive  - 15% HER2 positive | None reported | Arizona Activity Frequency Questionnaire to assess leisure-time PA (MET]-h/wk). | - Incidence of CVE decreased across increasing total MET-h/wk (Ptrend p<0 .001).  -Compared with < 2 MET-h/wk, adjusted HR: 0.91 (CI: 0.76 to 1.09) for 2 to 10.9 MET-h/wk.  -HR: 0.79 (CI 0.66 to 0.96) for 11 to 24.5 MET-h/wk.  -HR: 0.65 (CI: 0.53 to 0.80) for ≥ 24.5 MET-h/wk.  -Incidence of CAD and HF (each p<.05).  -Adherence to national exercise guidelines with cancer (≥ 9 MET-h/wk) was associated with an adjusted 23% reduction in the risk of CVE (p<.001). |

| **Trial** | **Methodology** | **Type of cancer** | **Sample size** | **Phase of treatment** | **Comorbidities** | **Exercise intervention** | **Outcome** |
| --- | --- | --- | --- | --- | --- | --- | --- |
| Palomo et al. 2017 [151]  Okwuosa et al. 2019 [149] | Prospective, World Health Initiative (WHI) | Breast | n=4015 | Nonmetastatic | 56% HT  46% BMI≥ 30, 8% DM | -self-report questionnaire assessing leisure-time physical activity  -MET h/week | -Incidence of composite CVE decreased across increasing total MET h/week (p= 0.016)  -Compared with <2.5 MET-hours/week HR: 0.80 (CI: 0.59 to 1.09) for 2.5 to <8.6MET.  -HR: 0.9 (CI: 0.64 to 1.17) for 8.6 to <18 MET h/week.  -HR: 0.63 (CI: 0.45-0.88) for ≥18 MET h/week. |
| Nagy et al. 2017 [146] | Prospective | Breast | n=55 | -Resected right-sided BC  -Chemotherapy naive | None | PA defined as intensive exercise program≥30 min  ≥4-5x/week vs. UC | -After 5 years onset of symptoms of HF were less frequently reported in PA than in UC (19% vs. 68%, p=0.002). |
| Hayes et al. 2017 [67] | Randomized, controlled trial | Breast | n=194 | 6 weeks post surgery | None | 8 months AET vs. UC | -Median follow-up of 101 months.  AET:  -Overall survival HR: 0.45 (CI: 0.21-0.97; p = 0.037)  - Disease-free survival HR: 0.66 (CI:0.38-1.17; p = 0.155). |
| Irwin et al. 2015 [82] | Randomized, controlled trial | Breast | n=121 | Cancer survivors under aromatase inhibitors |  | AET vs. UC for 12 months  -150 min/week AET  -supervised RT 2x/week | -AET was superior to UC in VO_2peak_: +1.5 vs. -0.4ml/kg/min, p<.001).  -Arthralgia improved in ET vs. UC (p<.001). |
| Jones et al. 2013 [86] | Randomized | Breast | 20 | Stage IIB–IIIC breast adenocarcinoma scheduled for first-line neoadjuvant chemotherapy  10 vs 30% HER2-positive | 40 vs. 10% HT | -Neoadjuvant doxorubicin–cyclophosphamide and AET(AC-AET), relative to AC alone  -AET: 3 supervised cycle ergometry sessions/week at 60% to 100% of VO_2 peak,_ 30 to 45 min/session, for 12 weeks. | -VO_2 peak_ increased from 19.5 ± 7.6 to 22.1 ± 7.0 ml/ kg/ min (p = 0.04) in the AC-AET group, whereas it decreased from 17.5 ±4.8 to 16.0± 4.0 ml/ kg/ min (p = 0.04) in the AC group.  -Between group difference of >4.1 ml/kg/min favoring the AC-AET group (p < 0.01). |
| **Trial** | **Methodology** | **Type of cancer** | **Sample size** | **Phase of treatment** | **Comorbidities** | **Exercise intervention** | **Outcome** |
| Courneya et al. 2013 [37] | Multicenter randomized trial | Breast | 301 | During chemotherapy | 6% smokers | -3x/week supervised AET from start to end of chemotherapy  -a standard dose of 25 to 30 min AET  -a higher dose of 50 to 60 minutes AET  -a combined dose of 50 to 60 minutes of AET and RT.  -Initial intensity was individualized but generally began at 55% to 60% of VO_2peak_ and progressed to 70% to 75% of VO_2peak_ by week 6. | All groups deteriorated from baseline to the end of chemotherapy with the lowest decline in the more intense regimen (VO_2peak_ -2.5ml/kg/min) compared to the standard (-3.4ml/kg/min) and combined groups (-3,6ml/kg/min) |
| Howden et al. 2019 [78] | Prospective randomized | Breast | 28 | -Early stage patients undergoing anthracycline chemotherapy  -14%, HER2 positive in exercise group | none | 4 months AET vs. UC  -2 supervised exercise sessions/week  each consisting of 30 min AET and 30 min RT.  -One unsupervised 30–60 min home-based AET session/week.  -2–10% increase in intensity per non-treatment week.  -During the treatment week intensity was reduced by 2–10% with no change in training volume | -Decreases in VO_2 peak_ during chemotherapy were attenuated with AET (15 vs. 4% reduction, p = 0.010).  -Fewer participants in AET met the functional disability criterion (VO_2peak_<18ml/kg/min) (7 vs. 50%, p = 0.01).  -Compared with the baseline, HR_max_ was higher and SV was lower after chemotherapy (p = 0.003 and p= 0.06).  -Reduction in resting LVEF (from 63 to 60%, p= 0.002) and an increase in troponin (from 2.9 to 28.5 ng/ml, p < 0.0001) occurred in AET.  -CO_peak_ was the strongest predictor of functional capacity (p=0.012) |
| **Trial** | **Methodology** | **Type of cancer** | **Sample size** | **Phase of treatment** | **Comorbidities** | **Exercise intervention** | **Outcome** |
| Haykowsky et al. 2009 [69] | Single-group, prospective | Breast | 19 | HER2-amplified or HER2-overexpressed resected invasive breast cancer eligible for trastuzumab therapy | -3 smokers  -1 diabetic | Supervised AET 3x /week during initial 4 months of trastuzumab therapy. -cycle ergometer, 30 to 60 min at HR equal to 60% to 90% of VO_2peak_. | -Resting end-diastolic (pre: 120 ± 23 ml versus post: 133 ± 16 ml) and end-systolic volumes (pre: 44 ± 12 ml versus post: 55 ± 11 ml) increased.  -mass (pre: 108 ± 21 g versus post: 114 ± 18 g) increased.  - EF (pre: 64% ± 4% versus post: 59% ± 4%) decreased from baseline to post-intervention (all p<0.05).  -Peak EF was lower after 4 months (pre: 79 ± 4 versus post: 76 ± 6%; p = 0.087). |
| Foulkes et al. 2023 [52] | Randomized controlled trial | Breast | 104 | Stage I to III breast cancer patients scheduled for anthracycline-based chemotherapy | HT (12 vs. 10%)  -DM 2% each  -Overweight (73 vs 60%) | -AET vs. UC for 12 months.  -3 to 4x/week using a -Nonlinear step periodization model.  -A progressive increase in exercise volume of ≈5% to 10% each week was prescribed until the week immediately after chemotherapy cycle.  -Week of chemotherapy was considered a “deloading” week, during which training intensity was reduced by ≈5%. | -Compared with UC, AET was associated with a net 3.5-ml/kg/min improvement in VO_2peak_ that coincided with greater CO, SV, and left and right ventricular EF reserve (P<.001).  -Troponin levels after chemotherapy increased less in AET compared to UC (p=0.002). |
| **Trial** | **Methodology** | **Type of cancer** | **Sample size** | **Phase of treatment** | **Comorbidities** | **Exercise intervention** | **Outcome** |
| Fung et al. 2017 [53] | Observational | Testicular | 952 | Testicular cancer survivors 1 year post chemotherapy. | 8.3% smokers, 42% overweight | None, observational, self-reported vs. age-matched sedentary controls | PA was protective against advere health outcomes (p<0.05) |
| Adams et al. 2017 [5] | Randomized controlled trial | Testicular | 63 | -Confirmed history of testicular cancer and no evidence of precancer CVD  -36.5% had received chemotherapy  -17.5% radiotherapy | -21% Obesity  -19% pre-HT  -8% smokers | -UC or 12 weeks of supervised HIIT  -4 HIIT intervals for 4 min each  -Progression from 75% to 95% of VO_2peak_  -HIIT intervals were separated by 3-min active recovery intervals (at 5%-10% below the VT) | -HIIT was superior to UC for improving VO_2peak_ (adjusted between-group mean difference, 3.7ml O_2_/kg/min; CI: 2.4-5.1 p<.001) and CVD risk (p= 0.011)  -HIIT reduced the prevalence of modifiable CVD risk factors by 20% compared with UC |
| Cormie et al. 2015 [36] | Randomized controlled | Prostate | 63 | Exercise was commenced within 10 days of first androgen deprivation therapy | 3% smokers | ET vs. UC  -3-month supervised AET (3x/week (20-30min at 70-85% of HR_max_) and RT (60-85% of 1RPM). -Intensity was increased according to individual response | -Significant between-group differences favouring AET for VO_2peak_ (1.1ml/kg/min, p=0.004), muscular strength (4.0–25.9 kg, p≤0.026), total cholesterol (–0.52, p=0.028), sexual function (15.2,p=0.028), fatigue (3.1, p=0.042), psychological distress (-2.2,p=0.045), social functioning (3.8, p=0.015), and mental health (3.6–3.8, p≤0.022). |
| Richman et al. 2011 [165] | Prospective | Prostate | 1455 | Clinically localized prostate cancer | Smokers<10% | -Activity questionnaire  -Vigorous activity ≥6 MET | -Men who walked briskly for ≥3 h/wk had a 57% lower rate of progression than men who walked at an easy pace <3 h/wk (HR: 0.43; 0.21–0.91; p= 0.03).  -Walking pace was associated with decreased risk of progression independent of duration (brisk vs. easy pace, HR: 0.52, CI: 0.29–0.91; ptrend = 0.01) |
| **Trial** | **Methodology** | **Type of cancer** | **Sample size** | **Phase of treatment** | **Comorbidities** | **Exercise intervention** | **Outcome** |
| Brown et al. 2018 [22] | Randomized trial | Colorectal | 39 | -Stage I to III  -completed surgical resection and adjuvant chemotherapy within 36 months of entering the study  -self-reported min/week of moderate or vigorous intensity PA using the Paffenbarger Physical Activity Questionnaire | -5% smokers  -33% HT  -13% diabetes  -10% CVD  74% in ECOG 0-2 | 2 types of AET (6 months) vs. UC  150 min/week (low-dose)  -300 min/week (high-dose)  -AET was performed for 6 months using in-home treadmills with heart rate monitoring | Compared with UC sICAM-1 decreased for 134.9 ng/ml (CI: 238.1 to 31.6) in the low-dose group, and 114.8 ng/ml (CI, 222.5 to 7.1) in the high-dose group. |
| Meyerhardt et al. 2006 [131] | Randomized trial | Colon | 832 | -Stage III, local cancer  -ECOG 0-2 | None reported | -Patients reported on PA approximately 6 months after completion of therapy and were observed for recurrence or death.  -Each activity on the questionnaire was assigned a MET task score | -Compared with ≤ 3 METh/week of PA, HR for disease-free survival was 0.51 (CI: 0.26 to 0.97) for 18 to 26.9 MET h/week and HR 0.55 (CI: 0.33 to 0.91) for 27 or more MET h/week.  -Postdiagnosis activity was associated with improvements in recurrence-free survival (p for trend p=0.03) and overall survival (p for trend p=0 .01). |
| Triguero-Canovas et al.2023 [197] | Randomized controlled trial | Colorectal | 60 | Prior to undergoing minimally invasive surgery | -43 HT  -16% diabetes  -16% smokers | -AET (Trimodal prehabilitation) vs. UC -Program with recommendations for PA, nutritional supplementation, and relaxation exercises during the preoperative period and during 6–8 weeks after surgery compared to UC.  -AET: daily AET and 3x/ week RT.  -AET individualized according to the physical condition | -AET reduced postoperative complications (17.4% vs. 33.3%, p = 0.22) and hospital stay (5.74 vs. 6.67 days, p = 0.30).  -6MWT showed a signifcant improvement in the prehabilitation group (+78.9 m).  -Six weeks after surgery, prehabilitation showed a signifcant improvement in the 6MWT (+68.9 m vs. −27.2 m, p = 0.01).  -Signifcant diferences were observed in ergospirometry between diagnosis and 6 weeks postoperatively (+0.79 METs vs. −0.84 METs, p = 0.001). |
| **Trial** | **Methodology** | **Type of cancer** | **Sample size** | **Phase of treatment** | **Comorbidities** | **Exercise intervention** | **Outcome** |
| Sandmael et al. 2017 [170] | Randomized | Head and neck | 50 | -Squamous cell carcinoma originating in the head and neck (nasopharynx, oropharynx, or hypo pharynx; larynx; and oral cavity, except T1N0M0 laryngeal cancer)  -Referral for curative radiotherapy with or without chemotherapy | -25 vs. 5% smokers  -50 vs. 47% pharyngeal cancer | -exercise and nutrition intervention during (EN-DUR) or after radiotherapy (EN-AF)  -6-week treatment period.  -2 progressive RT sessions/week, 30 min each, total of 12 sessions | -Total muscle mass reduction of -2.6 cm^2^ /m^2^ (p=0.002 in the EN-DUR and -2.3cm^2^/m^2^ (p=0.062) in the EN-AF group). |
| Samuel et al. 2019 [168] | Randomized | Head and neck | 148 | -Patients received radical radio-chemotherapy for HNC -conservative treatment  -ECOG<2 | Not reported | -AET/RT vs. UC 11 weeks (7 weeks in- and 4 weeks out of hospital)  -AET: 5 days/week (except on day of chemotherapy) at an intensity of 3–5/10 RPE on modified Borg’s scale  -AET: Brisk walking, 15–20 min  RT: Major muscles of upper limb and lower limb, 2 sets (1 set = 8 to 15 repetitions)  -UC: PA recommendations of 3 10-min walks during the day for 5 days/week. | -Improvement in functional capacity (p < 0.001), quality of life (p < 0.001), and prevention of worsening of fatigue (p < 0.001) in AET/RT |
| **Trial** | **Methodology** | **Type of cancer** | **Sample size** | **Phase of treatment** | **Comorbidities** | **Exercise intervention** | **Outcome** |
| Samuel et al. 2013 [169] | Randomized | Head and neck | 48 | -Patients under radiochemotherapy  -ECOG<2 | 56% smokers | -AET (6 weeks) vs. UC  -AET: Individualized and structured programme  -Brisk walking and an active ET.  -1st: Individually tailored brisk walking for 15 to 20 min at an RPE between 3-5/10 for 5 days/week for six weeks.  -2nd: Individually tailored active RT (3-5/10 RPE) for the major muscle groups of upper limb and lower limb, 5 days/ week for 6 weeks. | -6MWD improved in AET (42 m), while the control group showed a decrease of 96 m (p<0.05). |
| Allen et al. 2022 [8] | Randomized | Esophageal | 54 | Locally advanced, planned for neoadjuvant therapy plus esophagogastrectomy, or total gastrectomy | -11% smokers  - 2% stroke  4% CAD  -13% lung disease  -56% HT | -AET vs. UC  -Supervised ET for 1 h, 2x/week for 15 preoperative weeks. -Tailored program based on baseline CPET and HRR.  -20 min at intensities that increased from 40% to 60% HRR across the 15 weeks.  -RT involving six major muscle groups for two sets of 12 reptitions at RPE 12–14.  -Home-based exercise program for 1 h, 3x/week | -Attenuated peak VO_2_ decline in AET (-0.4, CI: -0.8 to 0.1 vs. -2.5, CI -2.8 to -2.2 ml/kg/ min, p = 0.022).  -Less skeletal muscle loss in AET [mean change in AET -11.6, CI -14.2 to -9.0 vs. UC -15.6, CI -18.7 to -15.4) cm2 /m2, p = 0.049].  -More AET completed neoadjuvant therapy at full dose (AET: 75% vs. 46% in UC, p = 0.036).  -No difference in median length of hospital stay (AET 11 vs 16 days in UC, p = 0.155) or 30-day readmission [ET 22% 23% in UC, p = 0.815). |
| **Trial** | **Methodology** | **Type of cancer** | **Sample size** | **Phase of treatment** | **Comorbidities** | **Exercise intervention** | **Outcome** |
| Bryant et al. 2018 [23] | Randomized | Acute leukemia | 18 | Newly diagnosed AML or ALL and admission to begin induction chemotherapy | Not reported | 4-week mixed-modality supervised AET (4 times a week, 2x/day) vs. UC.  - AET: Walking or stationary bike and  -RT: Use of different strengths of resistance bands.  -Progressive model consisted of 5 to 15 min AET and 10 to 20 minutes RT.  -Intensity progressed from 50% to 70% of HRR.  -RT intensity increased from lighter to heavier. | -No difference in 6MWD (AET/RT: +37.8m vs. UC: +13.7m, p=0.84).  -AET/RT showed a trend to less fatigue (p=0.11). |
| Courneya et al. 2009 [38] | Randomized | Hodgkin and non-Hodgin lymphoma | 122 | -Histologically confirmed  - chemotherapy or no treatment | -11% smokers  -29% physically active prior to AET  -29% HT | -UC or 3x/week for 12 weeks of supervised AET.  -Follow-up after 6 months  - Intensity began at 60% of baseline VO_2peak_, and increased by 5% each week to 75% by the fourth week.  -Duration began at 15 to 20 min for the first 4 weeks and increased by 5 min/ week to 40 to 45 min in the ninth week.  -One session per week: HIIT above the VT in week 7 and 9.  -UC were asked not to increase exercise | -AET was superior to UC for patient-rated physical functioning (mean group difference: 9.0, CI: 2.0 to 16.0, p =0.012), overall quality of life (p= 0.021), fatigue (p=0 .013), happiness (p=0 .004), depression (p=0.005), general health (p= .001), CRF (p =0.001), and lean body mass (p= 0.008) |

Appendix. Depiction of selected exercise trials across different cancer entities. AET: Aerobic exercise training. AML: Acute myeloid leukemia. ALL: Acute lymphocytic leukemia. BC: Breast cancer. BMI: body mass index. CPET: Cardiopulmonary exercise testing. CRF: Cardiorespiratory fitness. CVD: Cardiovascular disease. ECOG: Eastern Cooperative Oncology Group, ranging from 0-5, with 0 being fully active, and 5 being completely disabled. EF: Ejection fraction. FRS: Framingham risk score. CO_peak_: Peak cardiac output. CV: Cardiovascular event. DM: Diabetes. HF: Heart failure. HIIT: High intensity interval training. HR: Heart rate. HR_max_: Peak heart rate. HRR: Heart rate reserve. HT: Hypertension. MET: Metabolic equivalent. 1RPM: One repetition maximum. PA: Physical activity, which is, contrary to exercise training, not structured. RPE: Rate of perceived exertion. RT: Resistance training. 6MWD: 6 minute walk distance. SV: Stroke volume. UC: Usual care. VO_2peak_: Peak oxygen consumption. VT: Ventilatory threshold.
